# PURPOSE IN LIFE PROTECTS AGAINST COGNITIVE DECLINE AMONG OLDER ADULTS

**DOI:** 10.1093/geroni/igz038.2414

**Published:** 2019-11-08

**Authors:** Giyeon Kim, Su Hyun Shin, Monica Scicolone, Patricia A Parmelee

**Affiliations:** 1 Chung-Ang University, Seoul, Korea, Republic of; 2 The University of Alabama, Tuscaloosa, Alabama, United States

## Abstract

Objective: This study examined whether having a sense of purpose in life protects against cognitive decline among older adults and whether purpose in life moderates the relationship between selected risk factors (age, sex, and race/ethnicity) and cognitive abilities. Methods: This was a longitudinal analysis of existing secondary data of adults (N = 11,557) aged 50 or older using the 2006 −2012 waves of the Health and Retirement Study. The study measured purpose in life, cognitive functioning score, and various covariates. Results: Growth curve modeling revealed that, after adjusting for covariates, purpose in life was positively associated with participants’ total cognition scores. Purpose in life significantly moderated the relationship between age and race/ethnicity and cognitive decline. Further, purpose in life was a protective factor against cognitive decline for those who were older and black. There was no significant interaction between purpose in life and sex. Conclusion: Having a purposeful life protects against cognitive decline in older adults, and the associations varied by age and race/ethnicity, but not by sex. Potential ways to increase purpose in life are discussed in a clinical context.

